# High expression of Collagen Triple Helix Repeat Containing 1 (CTHRC1) facilitates progression of oesophageal squamous cell carcinoma through MAPK/MEK/ERK/FRA-1 activation

**DOI:** 10.1186/s13046-017-0555-8

**Published:** 2017-06-23

**Authors:** Chunni Wang, Zitong Li, Fei Shao, Xueying Yang, Xiaoli Feng, Susheng Shi, Yibo Gao, Jie He

**Affiliations:** 1 Department of Thoracic Surgery, National Cancer Center/Cancer Hospital, Chinese Academy of Medical Sciences and Peking Union Medical College, Beijing, 100021 China; 2 Department of Pathology, National Cancer Center/Cancer Hospital, Chinese Academy of Medical Sciences and Peking Union Medical College, Beijing, 100021 China

**Keywords:** Oesophageal squamous cell carcinoma (ESCC), Collagen triple helix repeat containing 1 (CTHRC1), Prognosis, Progression, ERK pathway, FRA-1, MMP14

## Abstract

**Background:**

Oesophageal cancer is one of the most common malignancies worldwide,and oesophageal squamous cell carcinoma (ESCC) is the predominant histological type both globally and in China. Collagen triple helix repeat containing 1 (CTHRC1) has been found to be upregulated in ESCC. However, its role in tumourigenesis and progression of ESCC remains unclear.

**Methods:**

Using our previous ESCC mRNA profiling data, we screened upregulated genes to identify those required for proliferation. Immunohistochemistry was performed to determine the level of CTHRC1 protein expression in 204 ESCC patients. Correlations between CTHRC1 expression and clinicopathological characteristics were assessed. In addition, pyrosequencing and 5-aza-dC treatment were performed to evaluate methylation status of CTHRC1 promoter. *In vitro* and *in vivo* analyses were also conducted to determine the role of CTHRC1 in ESCC cell proliferation, migration and invasion, and RNA sequencing and molecular experiments were performed to study the underlying mechanisms.

**Results:**

Based on mRNA profiling data, *CTHRC1* was identified as one of the most significantly upregulated genes in ESCC tissues (*n* = 119, fold change = 20.5, *P* = 2.12E-66). RNA interference screening also showed that CTHRC1 was required for cell proliferation. Immunohistochemistry confirmed markedly high CTHRC1 protein expression in tumour tissues, and high CTHRC1 expression was positively correlated with advanced T stage (*P* = 0.043), lymph node metastasis (*P* = 0.023), TNM stage (*P* = 0.024) and poor overall survival (*P* = 0.020). Promoter hypomethylation at cg07757887 may contribute to increased CTHRC1 expression in ESCC cells and tumours. Forced overexpression of CTHRC1 significantly enhanced cell proliferation, migration and invasion, whereas depletion of CTHRC1 suppressed these cellular functions in three ESCC cell lines and xenografts. CTHRC1 was found to activate FRA-1 (Fos-related antigen 1, also known as FOSL1) through the MAPK/MEK/ERK cascade, which led to upregulation of cyclin D1 and thus promoted cell proliferation. FRA-1 also induced snail1-mediated MMP14 (matrix metallopeptidase 14, also known as MT1-MMP) expression to facilitate ESCC cell invasion, migration, and metastasis.

**Conclusions:**

Our data suggest that CTHRC1 may act as an oncogenic driver in progression and metastasis of ESCC, and may serve as a potential biomarker for prognosis and personalized therapy.

**Electronic supplementary material:**

The online version of this article (doi:10.1186/s13046-017-0555-8) contains supplementary material, which is available to authorized users.

## Background

With an estimated 455,800 new cases and 400,200 deaths each year, oesophageal cancer is the sixth leading cause of cancer death and the eighth most common cancer worldwide [1]. Oesophageal squamous cell carcinoma (ESCC) is the predominant histological type both in China and around the world. Despite advancements in population screening and standardized multidisciplinary treatment over the last four decades [2], our previous report showed that ESCC remains the fourth leading cause of cancer-related death in China [3], with a dismal 5-year survival rate of only 20.9% [4]. The poor outcome of patients is primarily attributed to the high rate of ESCC metastasis, including both regional lymph node and further distant metastases [5]. Therefore, it’s vital to identify the underlying molecular mechanisms that drive progression, especially metastasis, of ESCC for predicting patients’ prognosis and improving rational design of personalized medicine.

Collagen triple helix repeat containing 1 (CTHRC1) is a secreted glycoprotein that can reduce collagen matrix deposition and promote the mobility of fibroblasts and smooth muscle cells [6–8]. Indeed, overexpression of CTHRC1, which has been reported in various malignancies, is suggested to serve as an independent prognostic factor [9–12]. Recently, a germline mutation in CTHRC1 gene was identified to be associated with Barrett’s oesophagus and oesophageal adenocarcinoma [13], and high CTHRC1 expression in ESCC was revealed by expression profiling studies involving a small number of cases [14, 15]. However, these findings should be confirmed in studies including larger groups and the cellular function and clinical implications of CTHRC1 in ESCC need to be resolved.

In this study, we sought to confirm in a much larger cohort aberrant elevated expression of CTHRC1 and to investigate its association with clinicopathological characteristics in ESCC. To assess the effect of CTHRC1 on malignant phenotypes of ESCC cells *in vitro* and *in vivo*, we then established multiple cell lines with stable depletion or overexpression of CTHRC1. Furthermore, we defined the underlying signalling pathways and transcription factors that depend on CTHRC1 activation and are responsible for ESCC progression.

## Methods

### Patients and tissue specimens

The study design and use of clinical samples were approved by the Ethics Committee of Cancer Hospital, Chinese Academy of Medical Sciences and Peking Union Medical College. A total of 204 formalin-fixed and paraffin-embedded (FFPE) ESCC tissue samples were obtained with informed consent and agreement from the biobank of Cancer Hospital of Chinese Academy of Medical Sciences. From 2000 to 2008, specimens were surgically resected from patients with stage I-III ESCC and who did not receive preoperative treatment. The clinicopathological characteristics of these patients are summarized in Table 1. Five tissue microarrays (TMAs) were constructed by incorporating one representative core of each tissue. The microarrays contained 204 primary ESCC tumour tissues, 169 of which were accompanied by adjacent non-tumour epithelial tissues.

**Table 1:** Correlations between CTHRC1 levels in ESCC tissues and clinicopathological characteristics of patients with ESCC

Characteristic		CTHRC1 expression		*P*
		Low	High	
Age	≤60	49	67	0.128
	>60	28	60	
Gender	Male	62	106	0.593
	Female	15	21	
Tobacco use	No	27	45	0.957
	Yes	50	82	
Alcohol use	No	30	46	0.695
	Yes	47	81	
Family history	No	65	106	0.858
	Yes	12	21	
Location	Upper/Middle	39	60	0.637
	Lower	38	67	
Histology grade	G1/G2	59	94	0.677
	G3	18	33	
T stage	T1/T2/T3	44	54	*0.043*
	T4	33	73	
Lymph node metastasis	No	49	60	*0.023*
	Yes	28	67	
TNM stage	I/II	39	44	*0.024*
	III	38	83	
FRA-1	Low	22	28	0.116
	High	45	97	
Snail1	Low	39	53	0.100
	High	33	73	
MMP14	Low	45	58	*0.022*
	High	26	67	
Cyclin D1	Low	53	66	*0.018*
	High	24	61	

χ 2test was used. *P* value was italicized when *P* < 0.05

*CTHRC1* Collagen triple helix repeat containing-1 (CTHRC1); *FRA*-*1* Fos-related antigen 1

### Immunohistochemistry and scoring

Immunohistochemistry (IHC) was performed as previously described [16], using anti-CTHRC1 (ab192778, Abcam, USA), anti-FRA-1 (TA500624S, Origene, USA), anti-cyclin D1 (2978, CST, USA), anti-snail1 (TA500316S, Origene, USA) and anti-MMP14 (ab51047, Abcam, USA) antibodies. Slides were evaluated independently by two pathologists (S.S. & X.F.). The staining intensity was graded as 0 (negative), 1 (low), 2 (moderate) or 3 (high), and the proportion of staining was evaluated as 0 (negative), 1 (<10%), 2 (10–50%), 3 (51–80%), or 4 (>80%). The intensity and proportion scores were multiplied to generate the IHC index. The expression level was considered as low (IHC index < 6), and as high (IHC index ≥ 6).

### Cell culture

All cell lines used in this study were regularly authenticated by short tandem repeat (STR) profiling. KYSE510, KYSE30, KYSE450, KYSE180 and KYSE70 cells were cultured in RPMI 1640 medium supplemented with 10% foetal bovine serum, 100 UI/ml penicillin and 100 UI/ml streptomycin (Gibco, USA). Het1a, a non-malignant immortalized human oesophageal squamous cell line, was cultured in BEGM (Bronchial Epithelial Cell Growth) medium (Lonza, USA). All cell lines were maintained in a humidified incubator at 37 °C and 5%CO2.

### Transfection and stable cell line establishment

Small interfering RNA (SiRNA; Dharmacon, USA) and plasmid transfections were performed using Lipofectamine RNAiMAX Transfection Reagent and Lipofectamine 3000 (Invitrogen, USA), respectively. For silencing of CTHRC1, two short hairpin RNA (shRNA) oligonucleotides (5’-GCTATCTGGGTTGGTACTTGTTTCAAGAGAACAAGTACCAACCCAGATAGCTT-3’ and 5’-GCTTCTACTGGATGGAATTCATTCAAGAGATGAATTCCATCCAGTAGAAGCTT-3’) were cloned into the pLKD-CMV-R&PR-U6-shRNA vector (Heyuan, China). The negative control (NC) sequence was 5’-TGTTCTCCGAACGTGTCACGTTTCAAGAGAACGTGACACGTTCGGAGAACTT-3’. For overexpression, the coding DNA sequence (CDS) of CTHRC1 was cloned into the pLenti-EF1a-EGFP-P2A-Puro-CMV-MCS vector (Heyuan, China); the empty vector was used as the negative control. Lentivirus packaging and purification and cell infection were carried out with ViraPower^TM^ Lentiviral Expression Systems (Invitrogen, USA) according to the manufacturer’s instructions. Cells were selected using medium containing 1.5 μg/ml puromycin (Sigma-Aldrich, USA). The efficiency of knockdown and overexpression were confirmed by real-time polymerase chain reaction (PCR) and western blot.

### RNA interference (RNAi) screening

KYSE30, KYSE510 and KYSE70 cells were plated in 96-well plates and transfected in triplicate with on-target plus smartpool siRNA (Dharmacon, USA). After 72 h, the cells were stained with 4’,6-diamidino-2-phenylindole (DAPI) (Sigma-Aldrich, Germany). Then the samples were imaged using a high content screening system (Operetta) and analysed using Harmony 3.1 software.

### Real-time PCR (RT-PCR)

RT-PCR was performed as previously described [17]. The primers used are listed in Additional file 1: Table S1.

### Western blot

Whole cell lysates were prepared using RIPA buffer supplemented with protease and phosphatase inhibitor cocktail (Thermo, USA) and culture supernatants were concentrated using Microcon centrifugal filters (Millipore, USA). Western blot was performed as previously described [17]. Primary antibodies against the following proteins were used: CTHRC1 (ab192778, Abcam, USA), p-c-Raf (9427, CST, USA), p-MEK1/2 (9154, CST, USA), p-ERK1/2 (4370, CST, USA), ERK1/2 (4695, CST, USA), p-FRA-1 (5841, CST, USA), FRA-1 (5281, CST, USA), cyclinD1 (2978, CST, USA), snail1 (3879, CST, USA), and MMP14 (13130, CST, USA). α − Tubulin (T9026, Sigma-Aldrich, USA) was used as a loading control.

### Cell proliferation and colony formation assays

Cell proliferation and colony formation assays were performed as previously described [18]. Cell proliferation was assessed using Cell Counting Kit-8 (CCK8). Images of the colony formation assay results were scanned and the clone number was determined using GeneSys software (Genecompany, China).

### Boyden chamber Transwell assay

For invasion and migration assays, we used 24-well Boyden chambers precoated with or without Matrigel matrix (Corning, USA), respectively. The experiments were performed as previously described [19].

### Xenograft model and lung metastasis model

All mice used in this study received humane care, and all animal experiments were performed in accordance with the guidelines approved by the Institutional Animal Care and Use Committee of Cancer Hospital, Chinese Academy of Medical Sciences and Peking Union Medical College. BALB/c-nu mice and non-obese diabetic (NOD)-SCID mice (female, 4–5 weeks old) were purchased from Huafukang (Beijing, China). For the xenograft model, shRNA- or vector-transfected KYSE510 cells and CTHRC1- or vector-transfected KYSE450 cells were injected into the right dorsal flanks of BALB/c-nu mice (5 × 10^6^ cells per animal, 8 mice per group). Tumour formation was monitored every 5 days by measuring tumour size with a calliper. The tumour volume was calculated using the formula: V = (L × W^2^)/2. After 4 weeks, all mice were sacrificed, and the tumours were excised and weighed. For the lung metastasis model, shRNA- or vector-transfected KYSE510 cells and CTHRC1- or vector-transfected KYSE450 cells were injected into NOD-SCID mice through the tail vein (1 × 10^6^ cells per animal, 8 mice per group). Ten weeks later, the mice were sacrificed, and the lungs were excised and fixed with Bouin’s solution followed by embedding in paraffin for haematoxylin and eosin (H&E) staining. The number of lung surface metastatic nodules was evaluated by gross and microscopic examination.

### RNA sequencing

RNA sequencing was performed using KYSE510-shCTHRC1 and KYSE510-vector cells. Total RNA extraction, quality analysis, cDNA library preparation and sequencing were performed at Novogene (Beijing, China). Raw RNA sequences were mapped to the GRCh37.hg19 genome based on TopHat and assembled using Cufflinks. Relative transcript levels are expressed as “fragments per kilobase of transcript per million mapped” (FPKM). Differentially expressed genes (DEGs) were identified using Cuffdiff. To verify the RNA sequencing data, we assessed the transcriptional level of twenty genes using RT-PCR (Additional file 2: Table S2).

### Pyrosequencing assay

The pyrosequencing assay was conducted by QIAGEN Translational Medicine Co., Ltd. (Suzhou, China). The primers used were as follows: F-5’-AGGATAGAGGGGGTTATAAAAAGA-3’ and R-5’- ACTCTAACACATTACAAAACCTTACA-3’.

### Statistical analysis

Statistical analyses were performed using Prism GraphPad version 6.0 (GraphPad Software Inc., San Diego, USA). Correlations between mRNA expression levels were analysed using Pearson’s correlation coefficient. A chi square test was performed to determine the association between clinicopathological variables and CTHRC1 expression. Survival analysis was carried out using a log-rank test. A Cox proportional hazards model was used to identify independent prognostic factors. The significance of differences between groups was analysed using two-tailed Student’s t-test and the results are expressed as the mean ± SD. Differences were considered significant when *P* < 0.05. *P < 0.05 and **P < 0.01.

## Results

### CTHRC1 is significantly overexpressed in ESCC cells and required for their proliferation

**Fig. 1:**
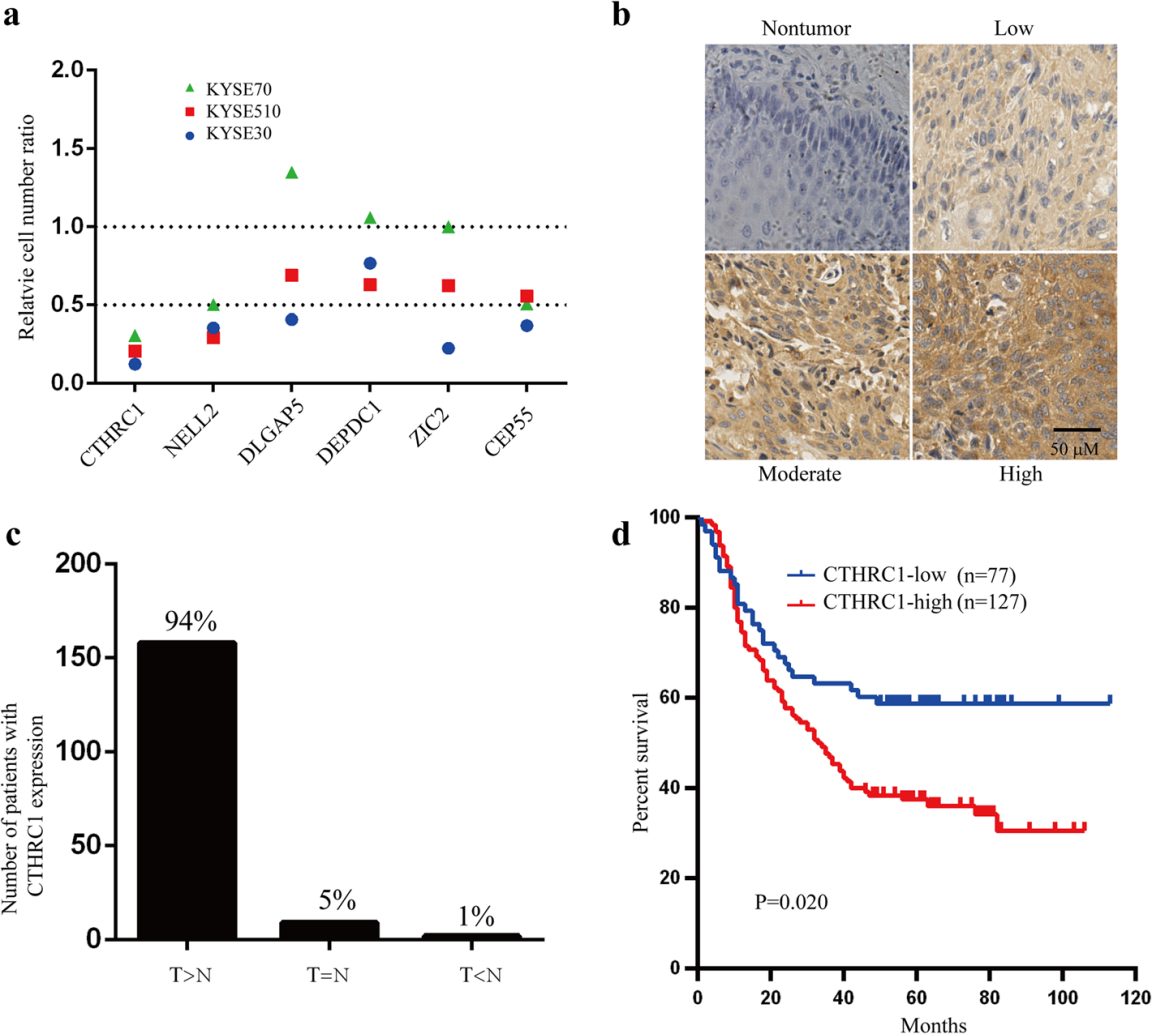
CTHRC1 is commonly upregulated in ESCC tissues and overexpression of CTHRC1 predicts poor prognosis. **a** Small RNA interference (RNAi) screening targeting Collagen triple helix repeat containing-1 (*CTHRC1*), Neural EGFL like 2 (*NELL2*), DLG associated protein 5 (*DLGAP5*), DEP domain containing 1 (*DEPDC1*), Zic family member 2 (*ZIC2*) and Centrosomal protein 55 (*CEP55*) in KYSE70, KYSE510 and KYSE30 cells. **b** Representative immunohistochemical (IHC) images of CTHRC1 staining in ESCC tumour tissues and non-tumour tissues. **c** The number and percent of patients with higher, equal or lower CTHRC1 staining in ESCC tumour tissues compared with non-tumour tissues. T: tumour tissue; N: non-tumour tissue. **d** Overall survival analysis based on the expression level of CTHRC1 measured by IHC in 204 ESCC patients. Survival rates were determined by the Kaplan-Meier survival analysis. *P* = 0.020, log-rank test

We previously conducted transcriptome-wide microarray profiling of 119 pairs of tumour and pair-matched non-tumour oesophageal mucosa samples [20]. Among the top 500 upregulated genes in tumor tissues, we selected *CTHRC1*, neural EGFL like 2 (*NELL2*), DLG associated protein 5 (*DLGAP5*), DEP domain containing 1 (*DEPDC1*), Zic family member 2 (*ZIC2*) and centrosomal protein 55 (*CEP55*) for this study (Additional file 3: Figure S1); these genes were also significantly upregulated in an independent gene expression profiling dataset [15]. The highest fold increase was found for *CTHRC1* (*n* = 119, fold change = 20.5, *P* = 2.12E-66, Wilcoxon test; false discovery rate (FDR) = 2.93E-64, Benjamin Hochberg procedure). RNAi screening targeting these genes in three ESCC cell lines was performed, and *CTHRC1* knockdown dramatically inhibited proliferation in all three cell lines (Fig. 1a).

Endogenous expression levels of *CTHRC1* in the ESCC cell lines were higher than in an immortalized oesophageal epithelium cell line (Additional file 4: Figure S2). IHC showed slight cytoplasmic staining of CTHRC1 protein in normal oesophageal epithelial cells, whereas moderate to strong staining in the cytoplasm and extracellular space was observed in most ESCC tumour tissues (Fig. 1b). Compared to matched non-tumor tissues, 94% (158/169) of tumour tissues exhibited stronger staining of CTHRC1 (Fig. 1c). Therefore, we focused on the role and mechanism of CTHRC1 in ESCC progression in this study.

### High expression of CTHRC1 in ESCC tumour tissue predicts poor prognosis

As CTHRC1 is almost universally overexpressed in tumour tissue compared to normal oesophageal epithelial tissue, we divided the sample set into two groups based on the CTHRC1 expression level (low or high) in tumour tissues and examined significant differences in clinicopathological characteristics between these two groups (Table 1). Notably, higher expression of CTHRC1 was significantly associated with advanced T stage (*P* = 0.043, chi square test), lymph node metastasis (*P* = 0.023, chi square test) and TNM stage (*P* = 0.024, chi square test). Patients exhibiting a high level of CTHRC1 expression had shorter overall survival than those with low CTHRC1 expression according to both Kaplan-Meier analysis (*P* = 0.020, log-rank test; Fig. 1d) and univariate Cox regression analysis (Table 2). However, CTHRC1 expression was not independently associated with overall survival by multivariate Cox regression analysis in this cohort after adjustment for age, histology grade, lymph node metastasis and TNM stage (Table 2).

**Table 2:** Univariate and multivariate analyses of overall survival among patients with ESCC

	Univariate analysis		Multivariate analysis	
	HR(95% CI)	*P*	HR(95% CI)	*P*
Age (≥60 *vs* <60)	1.958 (1.354–2.832)	*0.000*	1.676 (1.149–2.443)	*0.007*
Gender (Male *vs* Female)	1.178 (0.74–1.876)	0.490		
Tobacco use (Yes *vs* No)	0.869 (0.594–1.271)	0.470		
Alcohol use (Yes *vs* No)	0.890 (0.61–1.297)	0.544		
Family history (Yes *vs* No)	0.646 (0.369–1.130)	0.125		
Location (upper/middle *vs* lower)	0.782 (0.542–1.130)	0.191		
Histology grade (3 *vs* 1/2)	2.056 (1.39–3.041)	*0.000*	1.381 (0.919–2.076)	0.121
T stage (4 *vs* 1/2/3)	3.103 (2.085–4.620)	*0.000*	1.335 (0.766–2.326)	0.308
Lymph node metastasis (Yes *vs* No)	3.032 (2.055–4.474)	*0.000*	1.637 (1.043–2.570)	*0.032*
TNM stage (III *vs* I/II)	4.209 (2.672–6.632)	*0.000*	2.239 (1.119–4.482)	*0.023*
CTHRC1 (high *vs* low)	1.605 (1.074–2.399)	*0.021*	1.157 (0.768–1.743)	0.486

*P* value was italicized when *P* < 0.05

### Promoter hypomethylation may participate in upregulation of CTHRC1

**Fig. 2:**
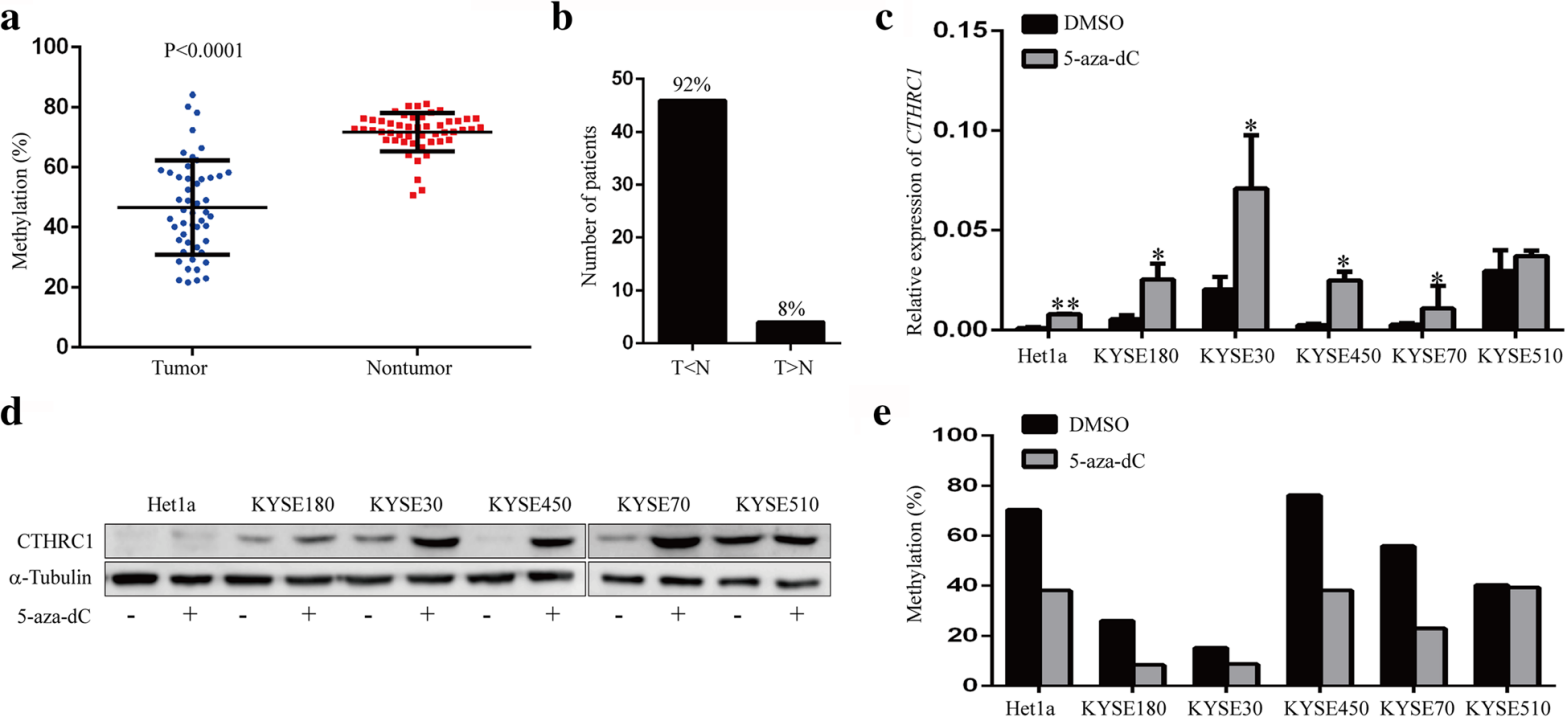
Promoter methylation is involved in regulating CTHRC1 expression in ESCC. **a** Promoter methylation of *CTHRC1* in paired tissue specimens from 50 ESCC patients was detected by pyrosequencing assay. *P* < 0.0001, paired two-tailed Student’s t-test. **b** The number and percent of patients with higher or lower promoter methylation of *CTHRC1* in ESCC tumour tissues compared with non-tumour tissues. T: tumour tissue; N: non-tumour tissue. **c** RT-PCR was performed to analyse the mRNA level of *CTHRC1* in ESCC cells treated or not with the demethylating agent 5-aza-dC (10 μM) for 72 h. The significance of difference between groups was analysed using two-tailed Student’s t-test. Data are presented as the mean (*n* = 3) ± SD. *P < 0.05 and **P < 0.01. **d** Western blot was performed to detect the protein level of CTHRC1 in culture supernatants of cells with or without 5-aza-dC treatment. **e** Promoter methylation of *CTHRC1* in ESCC cells with and without 5-aza-dC treatment was detected using a pyrosequencing assay

Previous genomic studies of ESCC didn’t show significant evidence of *CTHRC1* gene amplification [21]. We assessed whether promoter hypomethylation contributes to the elevated expression of *CTHRC1* in ESCC. Methylation array profiling of paired ESCC tissues revealed significantly lower methylation of cg07757887 (−1220 bp in the *CTHRC1* genomic region) in ESCC tumour tissues compared with non-tumour tissues (*n* = 67, ∆ß =−0.19; FDR = 1.34E-23, unpublished data). Pyrosequencing was performed for further validation, and as expected, cg07757887 methylation was significantly lower in ESCC tumour tissues compared with corresponding non-tumour tissues (*n* = 50, *P* < 0.0001, t-test, Fig. 2a), with 92% (46/50) of tumour tissues showing *CTHRC1* hypomethylation (Fig. 2b). With the exception of KYSE510 cells, treatment with the DNA methyltransferase inhibitor 5-aza-dC resulted in dramatically increased *CTHRC1* mRNA expression and protein production in five ESCC cell lines (Fig. 2c, d). Moreover, pyrosequencing confirmed distinctly increased methylation of cg07757887 in these cell lines except for KYSE510 cells (Fig. 2e), supporting the notion that *CTHRC1* expression may be closely associated with promoter methylation in ESCC.

### CTHRC1 promotes ESCC cell proliferation and tumour growth *in vitro* and *in vivo*

**Fig. 3:**
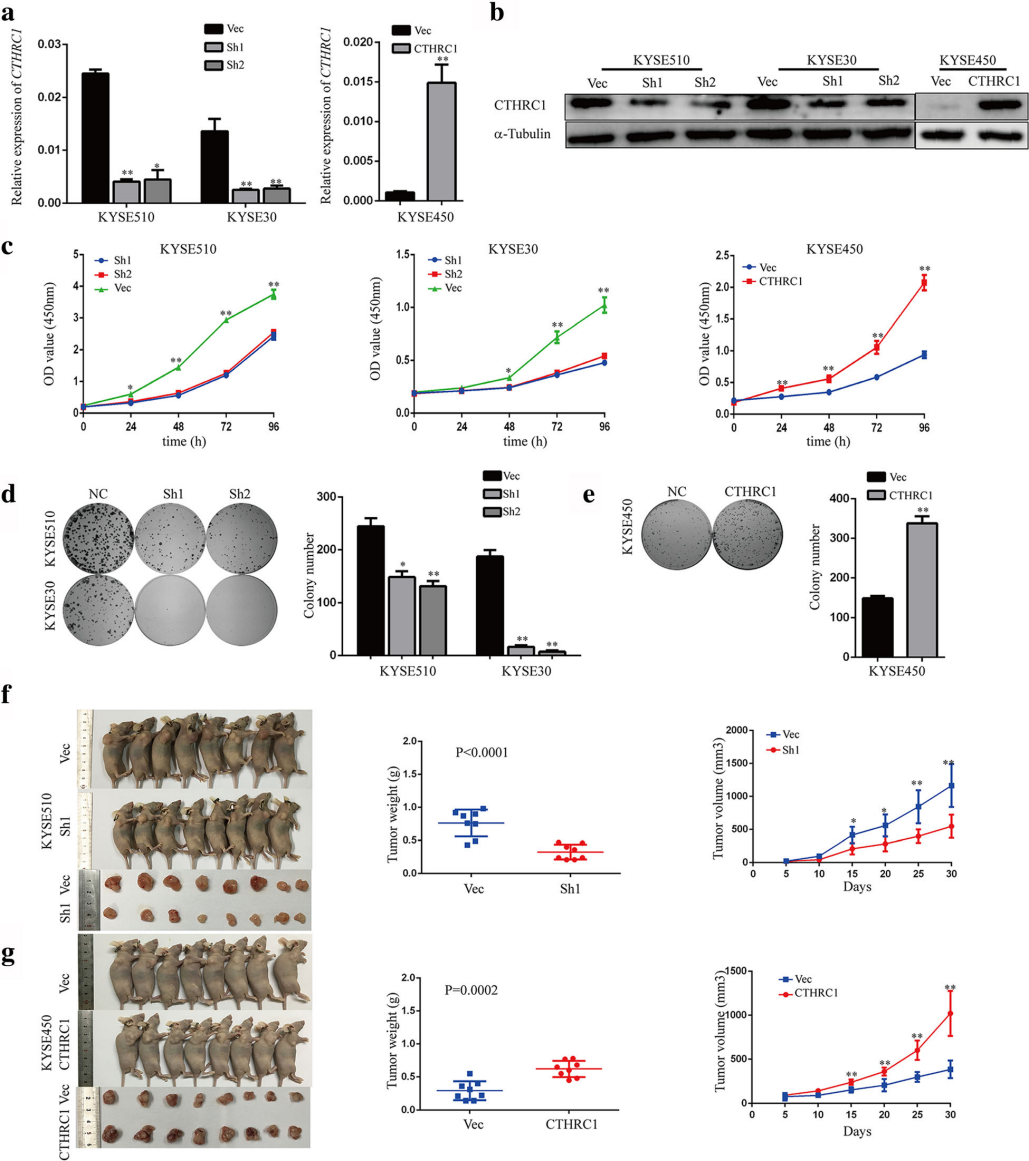
CTHRC1 is critical for ESCC cell proliferation and tumour growth*.* **a**, **b** Knockdown or overexpression efficiency of CTHRC1 in ESCC cells was verified by RT-PCR and western blot. **c** The proliferation capacity of KYSE510 and KYSE30 cells with depleted CTHRC1 expression and KYSE450 cells with enhanced CTHRC1 expression, as well as vector control cells was measured by CCK8 assay. **d**, **e** Representative images of colony formation assays using KYSE510, KYSE30 and KYSE450 cells (*left*). Columns show the mean number of clones formed in three independent experiments (*right*). **f**, **g** Representative images of tumour formation in nude mice subcutaneously inoculated with shCTHRC1- or vector-KYSE510 cells and CTHRC1- or vector-KYSE450 cells (*left*), and tumour weights and volumes for the two groups (*right*). The results are shown as the mean ± SD. The significance of the difference between groups was analysed using two-tailed Student’s t-test. *P < 0.05 and **P < 0.01

To investigate the effect of CTHRC1 on the malignant phenotypes of ESCC cells, we established cell models with CTHRC1 depletion or overexpression using three ESCC cell lines, and verified changes in expression by RT-PCR and western blot analyses (Fig. 3a, b). CTHRC1 depletion significantly attenuated cell proliferation and colony formation in KYSE510 and KYSE30 cells. Consistently, KYSE450 cells overexpressing CTHRC1 exhibited a significantly higher proliferation rate and colony formation capacity compared with KYSE450 cells transfected with the empty vector (Fig. 3c-e).

In agreement with the *in vitro* data, tumour size and weight were markedly reduced in the KYSE510-ShCTHRC1 group compared with the vector group (*P* < 0.0001, t-test, Fig. 3f), and KYSE450 cells with enhanced CTHRC1 expression formed significantly larger and heavier tumour xenografts compared to vector cells (*P* = 0.0002, t-test, Fig. 3g). Taken together, these results support the notion that CTHRC1 expression is critical for cell proliferation and tumour growth both *in vitro* and *in vivo*.

### CTHRC1 promotes migration and invasion of ESCC cells *in vitro* and *in vivo*

**Fig. 4:**
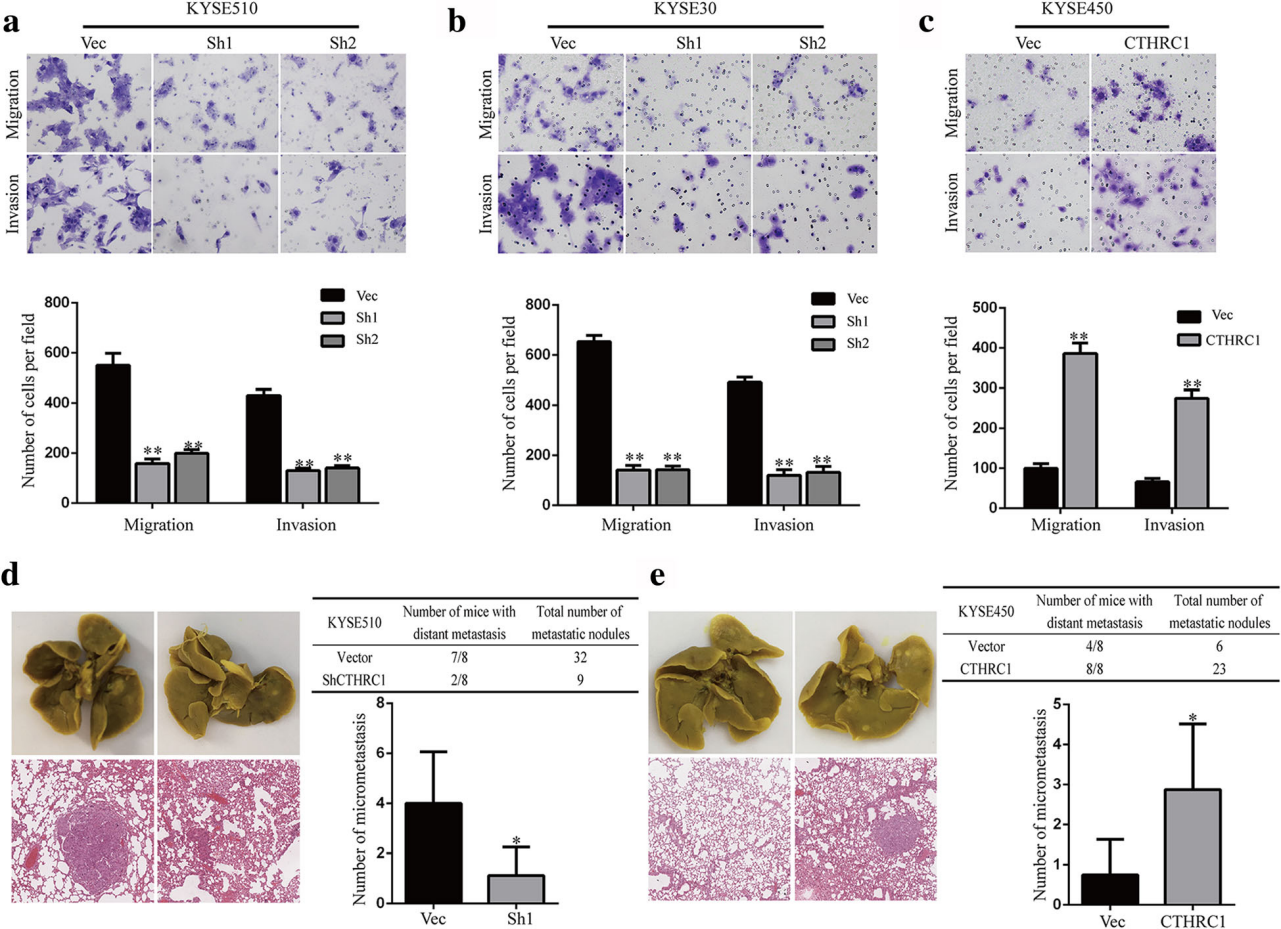
CTHRC1 facilitates migration and invasion of ESCC cells. Transwell assays were used to investigate migration and invasion of KYSE510 (**a**), KYSE30 (**b**) and KYSE450 (**c**) cells with altered CTHRC1 expression. **d**, **e** Representative images of isolated lungs (*upper*) and H&E-stained lung sections (*lower*) from each group. The number of mice with micrometastases and the total number of metastatic nodules of each group were also determined. Columns represent the mean number of metastatic nodules of each mouse. The results are shown as the mean ± SD. The *P* value was generated using two-tailed Student’s t-test. *P < 0.05 and **P < 0.01

To investigate the effect of CTHRC1 on the migration and invasion of ESCC cells, we conducted Boyden chamber Transwell assays. Overall, compared to cells transfected with the empty vector, migratory and invasive capacities were significantly suppressed in KYSE510 and KYSE30 cells with CTHRC1 knockdown (Fig. 4a, b) and remarkably enhanced in KYSE450 cells with CTHRC1 overexpression (Fig. 4c).

A lung metastasis model in NOD-SCID mice constructed via tail vein injection of cells showed a significantly lower incidence of and fewer pulmonary metastasis nodules in mice with KYSE510-ShCTHRC1 cell injection than in the control group (Fig. 4d). On the other hand, the incidence and number of pulmonary metastatic nodules in mice with KYSE450-CTHRC1 cell injection were higher than in the control group (Fig. 4e).

### CTHRC1 facilitates ESCC cells aggressiveness primarily via activation of the MAPK/MEK/ERK pathway

**Fig. 5:**
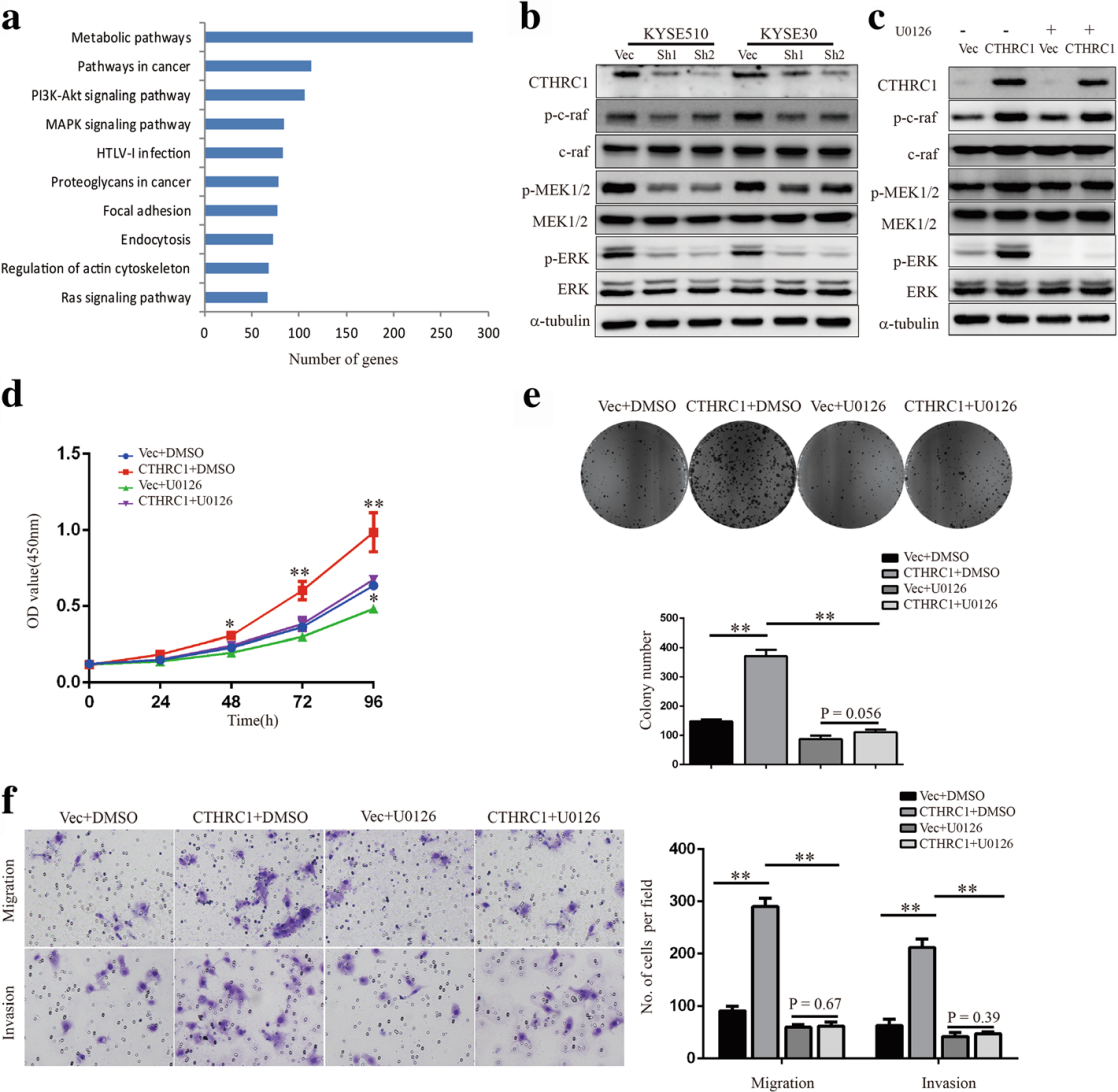
CTHRC1 exerts oncogenic functions by activating the MAPK/MEK/ERK pathway in ESCC cells. **a** Top-ranked KEGG pathway terms using DAVID. **b** Western blot was conducted to detect the protein levels of CTHRC1, c-raf, MEK1/2, ERK1/2 and phosphorylation of c-raf, MEK1/2, ERK1/2 in CTHRC1-knockdown KYSE510 and KYSE30 cells and corresponding vector control cells. **c** Western blot was conducted to detect the protein levels of CTHRC1, c-raf, MEK1/2, ERK1/2 and phosphorylation of c-raf, MEK1/2, ERK1/2 in CTHRC1-overexpressing KYSE450 cells and vector control cells. **d-f** KYSE450 with enhanced CTHRC1 expression and vector control cells were treated with the MEK inhibitor U0126 (10 μM) or dimethyl sulfoxide (DMSO). **d** Cell viability was measured using the CCK8 assay. **e** Colony formation assays were performed to measure the clonogenic capacity of cells. **f** Migration and invasion of cells were investigated using transwell assays. The results are shown as the mean ± SD. The difference between groups was analysed using two-tailed Student’s t-test. *P < 0.05 and **P < 0.01

We next explored the downstream signalling pathways responsible for CTHRC1-mediated ESCC cell aggressiveness using RNA sequencing with KYSE510 cells carrying ShCTHRC1 or the empty vector. A total of 3430 significantly upregulated (more than 2-fold) and 4377 downregulated (less than 50%) genes were selected for Kyoto Encyclopedia of Genes and Genomes (KEGG) pathway analysis [22]. The results indicated the PI3K-Akt and MAPK pathways as the top two pathways most significantly affected by CTHRC1 knockdown (Fig. 5a).

Western blot verified that Akt phosphorylation was decreased in CTHRC1-depleted KYSE510 and KYSE30 cells, and increased in CTHRC1-overexpressing KYSE450 cells, but the changes were relatively minor (Additional file 5: Figure S3). We observed phosphorylation of core members of the classical MAPK pathway, c-raf, MEK1/2 and ERK1/2, to be strongly decreased in KYSE510-ShCTHRC1 cells and KYSE30-ShCTHRC1 cells, and increased in KYSE450-CTHRC1-overexpressed cells compared with their control cells (Fig. 5b, c). Furthermore, treatment with a MEK1/2 inhibitor (U0126 at 10 μM) significantly reversed CTHRC1-induced proliferation, migration and invasion of KYSE450 cells (Fig. 5d-f), indicating that MAPK/MEK/ERK activation may underlie the phenotypes induced by CTHRC1 in ESCC cells.

### FRA-1 is the principle effector mediating activation of MAPK/MEK/ERK by CTHRC1 and upregulation of cyclin D1 and snail1/MMP14 expression

**Fig. 6:**
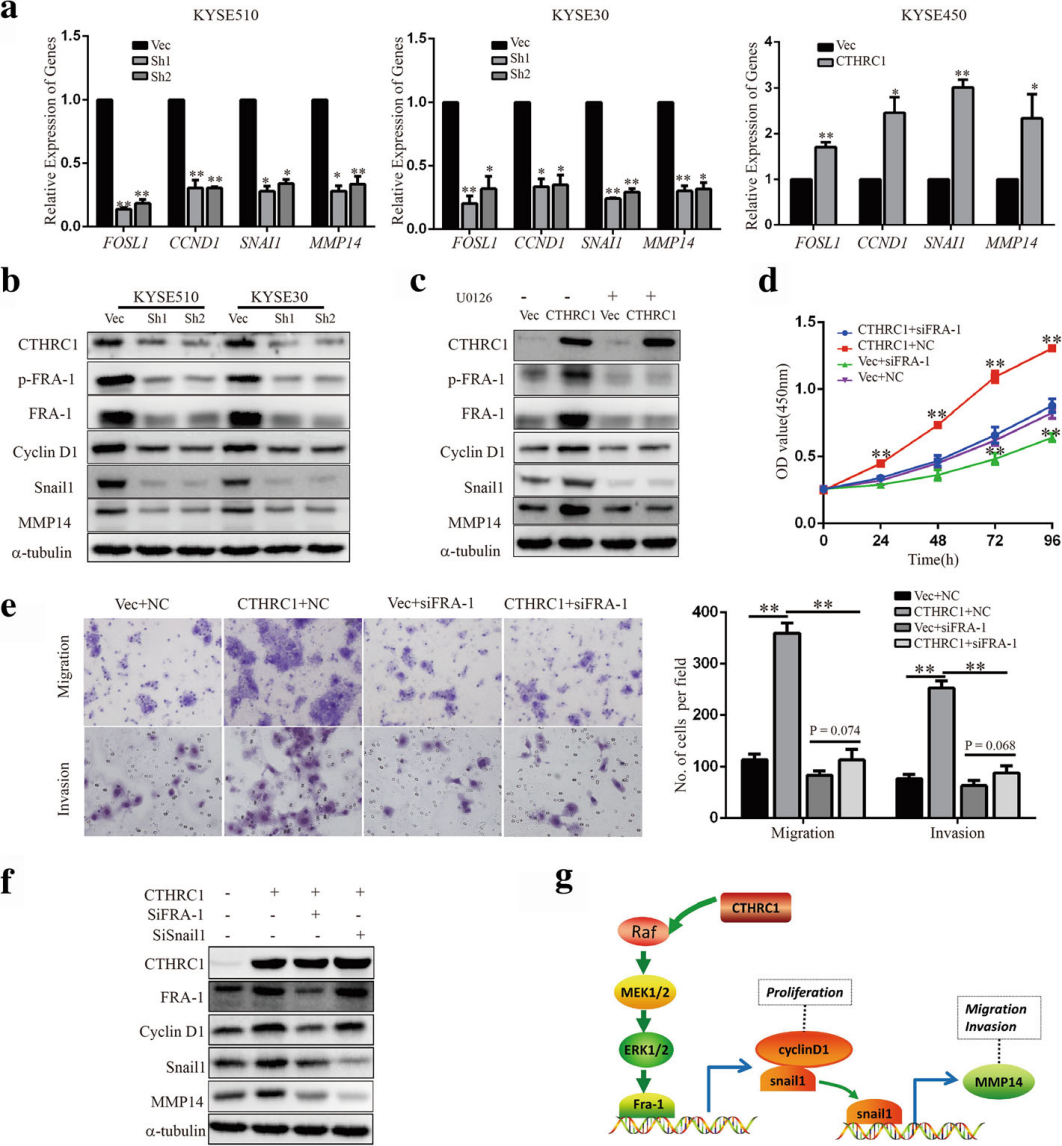
CTHRC1 upregulates cyclin D1 and snail1/MMP14 by activating the MAPK/MEK/ERK/FRA-1 cascade. **a** The relative mRNA levels of *FOSL1*, *CCND1*, *SNAI1* and *MMP14* in CTHRC1-knockdown cells or CTHRC1-overexpressing cells and control cells were detected by RT-PCR. **b** Western blot was conducted to detect the protein levels of CTHRC1, p-FRA-1, FRA-1, cyclin D1, snail1 and MMP14 in CTHRC1-knockdown KYSE510 and KYSE30 cells and corresponding vector control cells. **c** CTHRC1-overexpressing KYSE450 cells and control cells were treated with U0126 (10 μM) or DMSO. The protein levels of CTHRC1, p-FRA-1, FRA-1, cyclin D1, snail1 and MMP14 were detected by western blot. **d, e** CTHRC1-overexpressing KYSE450 cells and control cells were transfected with FRA-1 siRNA or negative control (NC) siRNA. **d** Cell viability was measured using the CCK8 assay. **e** Migration and invasion of cells were investigated using transwell assays. The results are shown as the mean ± SD. The difference between groups was analysed using two-tailed Student’s t-test. *P < 0.05 and **P < 0.01. **f** CTHRC1-overexpressing KYSE450 cells were transfected with FRA-1 siRNA or snail1 siRNA. The protein levels of CTHRC1, FRA-1, cyclin D1, snail1 and MMP14 in KYSE450 cells with different treatment were detected. **g** Schematic diagram illustrating the proposed CTHRC1-mediated activation of ERK1/2 signalling pathway and its role in ESCC cells

RNA sequencing analysis revealed *FOSL1* (Fos-related antigen 1, also known as FRA-1), an extensively studied MAP Kinase target [23–25], to be among the most significantly downregulated genes (80% off); *CCND1* (cyclin D1) (90% off), *SNAI1* (snail1) (60% off) and a known target of snail1, *MMP14* (matrix metallopeptidase 14, also known as MT1-MMP) (90% off) [26–28], were also significantly downregulated. Their dependency on CTHRC1 was repeatedly confirmed by RT-PCR and western blot in KYSE510 and KYSE30 cells with depleted CTHRC1 expression and in KYSE450 cells with CTHRC1 overexpression (Fig. 6a-c). Furthermore, administration of the MEK1/2 inhibitor U0126 abolished the increased phosphorylation of FRA-1 and increased protein levels of FRA-1, cyclin D1, snail1, and MMP14 induced by enhanced expression of CTHRC1 in KYSE450 cells (Fig. 6c).

Moreover, knockdown of FRA-1 using siRNA reversed the promotion of proliferation, migration and invasion by CTHRC1 (Fig. 6d, e), and western blot showed that knockdown of FRA-1 attenuated the upregulation of cyclin D1, snail1, and MMP14 in KYSE450 cells induced by CTHRC1 overexpression. Additionally, knockdown of snail1 reversed the increased expression of MMP14 induced by CTHRC1 (Fig. 6f), indicating that cyclin D1 and snail1 were downstream effectors of FRA-1; in turn, snail1 induced high expression of MMP14 in CTHRC1-overexpressing ESCC cells (Fig. 6g).

**Fig. 7:**
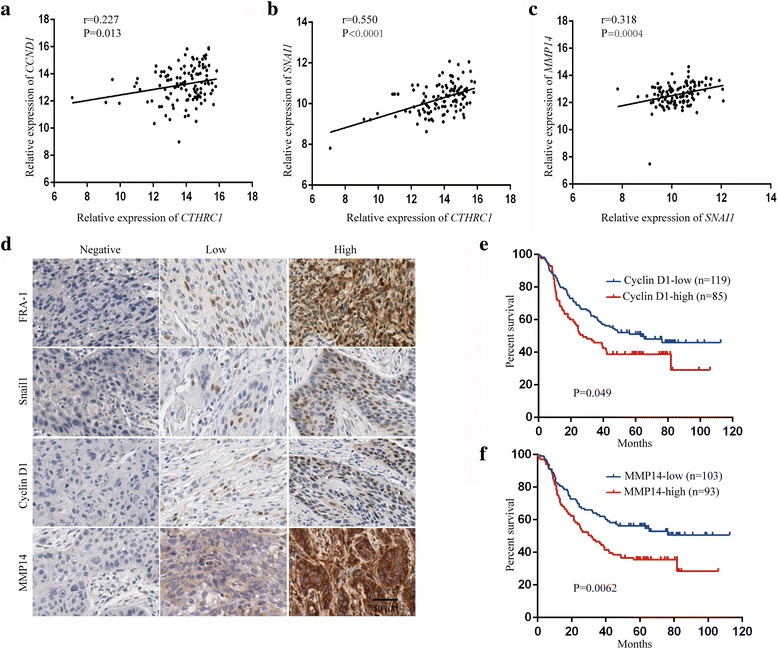
mRNA and protein level of CTHRC1, FRA-1, cyclin D1, snail1 and MMP14 in ESCC tissues. **a** Correlation between *CTHRC1* and *CCND1* according to transcriptome-wide microarray profiling data (*n* = 119). Pearson’s correlation coefficient. **b** Correlation between *CTHRC1* and *SNAI1* according to transcriptome-wide microarray profiling data (*n* = 119). Pearson’s correlation coefficient. **c** Correlation between *SNAI1* and *MMP14* according to transcriptome-wide microarray profiling data (*n* = 119). Pearson’s correlation coefficient. **d** Representative IHC images of FRA-1, snail1, cyclin D1 and MMP14 staining in ESCC tumour tissues. **e**, **f** Overall survival analysis based on the level of cyclin D1 and MMP14 expression, as measured by IHC, in ESCC patients. Survival rates were determined using Kaplan-Meier survival analysis

Consistent with the above *in vitro* studies, the level of *CTHRC1* mRNA was significantly positively correlated with those of *CCND1* and *SNAI1* in ESCC tumour tissues (*n* = 119, *r* = 0.227, *P* = 0.013; *r* = 0.550, *P* < 0.0001, Pearson’s correlation coefficient; Fig. 7a,b). Significant positive correlation between *SNAI1* and *MMP14* mRNA levels was also found (*r* = 0.318, *P* = 0.0004, Pearson’s correlation coefficient; Fig. 7c). Moreover, expression of CTHRC1 was positively associated with that of cyclin D1, as well as MMP14, at the protein level (*n* = 204, *P* = 0.018, chi square test; *P* = 0.022, chi square test; Table 1). In addition, Kaplan-Meier analysis revealed a significant association between shorter overall survival in ESCC patients and high expression of cyclin D1 (*n* = 204, *P* = 0.049, log-rank test, Fig. 7d), or MMP14 (*n* = 196, *P* = 0.0062, log-rank test; Fig. 7e).

## Discussion

This is the first study to present a comprehensive set of clinical and experimental evidence establishing CTHRC1 as an oncogenic factor that facilitates ESCC tumour progression and metastasis, resulting in poor prognosis. These data indicate that CTHRC1 may serve as a potential prognostic biomarker and treatment target in ESCC.

We also investigated the possible regulation mechanism of *CTHRC1* in ESCC. Treatment with a demethylation agent (5-aza-dC) markedly elevated CTHRC1 expression in most ESCC cell lines, which was in agreement with previous reports [16, 29, 30]. A pyrosequencing assay revealed a CpG site (cg07757887, -1220 bp in the *CTHRC1* genomic region) hypomethylated in ESCC tumour tissues, which has not been previously reported as being related to cancer. Although demethylation of the *CTHRC1* genomic region (-391 to +4 bp) in gastric cancer cells [30], in the first exon in colon cancer [16], and at -628 to -269 of the promoter region in hepatocellular carcinoma [29] has been reported, we did not find significant demethylation at those CpG sites in ESCC tumour tissues. Therefore, methylation of the CpG site involved in regulating *CTHRC1* may vary in different types of cancer.

Previous reports have suggested that other mechanisms may be involved in regulation of CTHRC1, such as TGF-β and Wnt3a pathway activation in gastric and oral squamous cell carcinoma, respectively [30, 31]. In addition, CTHRC1 was reported to be regulated by microRNA and long noncoding RNAs, such as let-7b and MALAT-1 [32, 33], which might explain the oncogenic role of MALAT-1 in ESCC [34]. Evidence to date supports the hypothesis that CTHRC1 integrates multiple pro-aggressiveness signalling pathways.

We also reveal for the first time that CTHRC1 exerts its effect on ESCC progression mainly through the Raf/MEK/ERK pathway, with dependence on the induction and activation of FRA-1, a FOS family transcription factor that binds to JUN-family proteins to form the AP-1 complex [35]. Transcriptional induction and post-translational stabilization of FRA-1 via MEK/ERK signalling increases the abundance of FRA-1, which has been causally linked to more aggressive behaviours of multiple cancer cell types [36–40], but not through a CTHRC1-dependent pathway.

There has been accumulating evidence for the significant role of MEK/ERK pathway in cancer development [41–44]. In accordance with the results of our study, the MEK/ERK pathway has been related to CTHRC1 in pancreatic cancer, without identification of any downstream effectors [45]. Another study suggested that CTHRC1 upregulated MMP9 via ERK activation in colorectal cancer [16]; however, alteration in *MMP9* expression was not indicated in our RNA sequencing data. Through transcriptome sequencing and extensive step-by-step *in vitro* analyses, we identified Cyclin D1 and snail1 as major downstream effectors of FRA-1, accounting for the CTHRC1-mediated regulation of proliferation and motility in ESCC cells. The most prominent function of snail1 in cancer cells is to induce the epithelial-mesenchymal transition (EMT) [46, 47], and it was recently reported that CTHRC1 upregulated snail1 to induce EMT by activating the Wnt/β-catenin signalling pathway in epithelial ovarian cancer [48]. Interestingly, we did not observe any meaningful alteration in β-catenin expression or in hallmarks of EMT [49], namely, E-Cadherin and vimentin, after CTHRC1 knockdown in ESCC cell lines (Additional file 5: Figure S3), suggesting that an alternative hypothesis is needed to explain findings for ESCC. Indeed, a few recent studies invoked other possible mechanisms by which snail1 could regulate cell migration and invasion, such as MMP14-mediated pro-invasive and metastatic activities [26–28]. However, the respective upstream mechanisms were not elucidated. Here, we not only show that MMP14 can be upregulated by snail1 activation, but also demonstrate it under regulation of CTHRC1/MAPK/MEK/ERK/FRA-1 signalling in ESCC.

It should be acknowledged that there was one limitation related to this study: we did not clarify how CTHRC1 activates the MAPK/MEK/ERK pathway. It was recently demonstrated that EGFR inhibitors attenuated the promoting effect of CTHRC1 on epithelial ovarian cancer invasion and that phosphorylation of EGFR and ERK1/2 was reduced in CTHRC1-silenced ovarian cancer cells [50]. Since CTHRC1 is a secreted protein, it is worth investigating in future studies whether CTHRC1 acts as a ligand of EGFR to activate the MAPK/MEK/ERK pathway in ESCC.

## Conclusions

In summary, our findings reveal that CTHRC1 plays a pivotal oncogenic role in ESCC proliferation, invasion, and metastasis by upregulating cyclin D1, snail1 and MMP14 through the Raf/MEK/ERK/FRA-1 pathway. Patients with high expression of CTHRC1 are possible candidates for biologic agents that affect the oncogenic circuit we found in ESCC, such as MEK1/2 inhibitors and CDK inhibitors. Additionally, the newly elucidated clinical implications of CTHRC1 in our cohort support its use as a potential prognostic marker for ESCC patients.

## Additional files

Additional file 1: Table S1. Oligonucleotide primers used for RT-PCR. (DOCX 16 kb)

Additional file 2: Table S2. Verification of RNA-Seq data by RT-PCR. (DOCX 16 kb)

Additional file 3: Figure S1. Analysis of Collagen triple helix repeat containing-1 (*CTHRC1*), Neural EGFL like 2 (*NELL2*), DLG associated protein 5 (*DLGAP5*), DEP domain containing 1 (*DEPDC1*), Zic family member 2 (*ZIC2*) and Centrosomal protein 55 (*CEP55*) mRNA levels according to previous transcriptome-wide microarray profiling data (*n* = 119). The *P* value was generated by Wilcoxon test. FC: Fold change. (TIF 554 kb)

Additional file 4: Figure S2. Expression of CTHRC1 in ESCC cell lines was analysed by RT-PCR (a) and western blot analysis (b). (TIF 488 kb)

Additional file 5: Figure S3 The levels of p-Akt, Akt, β-catenin, E-cadherin and vimentin proteins were determined by western blot in KYSE510 and KYSE30 cells depleted for CTHRC1 expression and KYSE450 cells overexpressing CTHRC1 as well as corresponding control cells. (TIF 670 kb)

## Abbreviations

BEGM: Bronchial epithelial cell growth
CDS: Coding DNA sequence
CEP55: Centrosomal protein 55
CTHRC1: Collagen triple helix repeat containing-1
DAPI: 4’,6-diamidino-2-phenylindole
DEG: Differentially expressed gene
DEPDC1: DEP domain containing 1
DLGAP5: DLG associated protein 5
ESCC: Oesophageal squamous cell carcinoma
FFPE: Formalin-fixed and paraffin-embedded
FPKM: Fragments per kilobase of transcript per million mapped
FRA-1: Fos-related antigen 1
H&E: Haematoxylin and eosin staining
IHC: Immunohistochemistry
MMP14: Matrix metallopeptidase 14
NC: Negative control
NELL2: Neural EGFL like 2
RNAi: RNA interference
RT-PCR: Real-time polymerase chain reaction
STR: Short tandem repeat
TMA: Tissue microarray
ZIC2: Zic family member 2
