# Supplementary material for: High expression of Collagen Triple Helix Repeat Containing 1 (CTHRC1) facilitates progression of oesophageal squamous cell carcinoma through MAPK/MEK/ERK/FRA-1 activation
Source: J Exp Clin Cancer Res. 2017 Jun 23;36:84. doi: 10.1186/s13046-017-0555-8 (PMC5481965; doi:10.1186/s13046-017-0555-8)
Supplement: Supplementary file 1 — Oligonucleotide primers used for RT-PCR. (DOCX 16 kb) [file 13046_2017_555_MOESM1_ESM.docx]

**Table S1. Oligonucleotide primers used for RT-PCR**

| genes | Forward primer sequence | Reverse primer sequence |
| --- | --- | --- |
| *CTHRC1* | 5'-TCATCGCACTTCTTCTGTGGA-3' | 5'-GCCAACCCAGATAGCAACATC-3' |
| *GAPDH* | 5'-CCTGGTATGACAACGAATTTG-3' | 5'-CAGTGAGGGTCTCTCTCTTCC-3' |
| *FOSL1* | 5'-CCGCCCTGTACCTTGTATCT-3' | 5'-CTGCTGCTACTCTTGCGATG-3' |
| *CCND1* | 5'-CCCTCGGTGTCCTACTTCAA-3' | 5'-CTCCTCGCACTTCTGTTCCT-3' |
| *SNAI1* | 5'-CGGAAGCCTAACTACAGCGA-3' | 5'-CTGACAGGGAGGTCAGCTCT-3' |
| *MMP14* | 5'-GTGACGGGAACTTTGACACC-3' | 5'-TTTGCCATCCTTCCTCTCGT-3' |
| *ACTN1* | 5'-CCAGGAGCAGATGAATGA | 5'-ATGAGGCAGGCTTTGAAC-3' |
| *AKTIP* | 5'-AACCAGTCCTCCACGAAC-3' | 5'-TCCAGGTAGAAGGGTCCA-3' |
| *CAV1* | 5'-AGAACCAGAAGGGACACAC-3' | 5'-AGAGAATGGCGAAGTAAATG-3' |
| *EIF4A1* | 5'-GGAACGAGAGGAGTGGAA-3' | 5'-ATGGCGGATACAGTGAAA-3' |
| *EIF4A3* | 5'-GGAGAGGGAAGAGTGGAA-3' | 5'-TTGAGGATACAGTGAAGTTGG-3' |
| *ERBB3* | 5'-GAGAGGTGTGAGGTGGTG-3' | 5'-GGGCAATGGTAGAGTAGAGA-3' |
| *ESR1* | 5'-CCTGATGATTGGTCTCGT-3' | 5'-CCCTCTACACATTTTCCCT-3' |
| *ITGB4* | 5'-GCTTCACACCTATTTCCCT-3' | 5'-ACCCAGTCCTCGTCTTCT-3' |
| *MTA1* | 5'-ATCTCCAGCACCCTCATC-3' | 5'-TCTTCTATTTCCCCTTCCTC-3' |
| *NEDD9* | 5'-AGGGTAAGGAGGAGTTTGAG-3' | 5'-CTGTTTGTGGTGGGTAGG-3' |
| *PEAK1* | 5'-AGACGCCAAAGGAGAAAC-3' | 5'-GCACAAAATCAGCCACAG-3' |
| *PTK2* | 5'-TCCAGTAAAATCCAGCCA-3' | 5'-TGTGCCATCTCAATCTCTC-3' |
| *RAP1B* | 5'-TTTATTCCATCACAGCACAG-3' | 5'-CCTTCCCTACAACTCTTTCA-3' |
| *TGIF1* | 5'-CGTTACAATGCCTATCCTTC-3' | 5'-CTTTGCCATCCTTTCTCA-3' |
| *TUBB6* | 5'-CTTCCTGCACTGGTTCAC-3' | 5'-CCTCTTCCTCATCCTCAAA-3' |
