# Supplementary material for: High expression of Collagen Triple Helix Repeat Containing 1 (CTHRC1) facilitates progression of oesophageal squamous cell carcinoma through MAPK/MEK/ERK/FRA-1 activation
Source: J Exp Clin Cancer Res. 2017 Jun 23;36:84. doi: 10.1186/s13046-017-0555-8 (PMC5481965; doi:10.1186/s13046-017-0555-8)
Supplement: Supplementary file 2 — Verification of RNA-Seq data by RT-PCR. (DOCX 16 kb) [file 13046_2017_555_MOESM2_ESM.docx]

**Table S2. Verification of RNA-Seq data by RT-PCR**

| Gene ID | Gene Symbol | RNA-seq FC | RT-PCR FC |
| --- | --- | --- | --- |
| 87 | *ACTN1* | 0.03 | 0.75 |
| 64400 | *AKTIP* | 0.03 | 0.43 |
| 857 | *CAV1* | 0.16 | 0.48 |
| 595 | *CCND1* | 0.11 | 0.32 |
| 115908 | *CTHRC1* | 0.46 | 0.02 |
| 1973 | *EIF4A1* | 0.09 | 0.45 |
| 9775 | *EIF4A3* | 0.08 | 1.11 |
| 2065 | *ERBB3* | 0.08 | 0.86 |
| 2099 | *ESR1* | 0.03 | 0.53 |
| 3691 | *ITGB4* | 0.04 | 0.71 |
| 9112 | *MTA1* | 0.11 | 0.56 |
| 4739 | *NEDD9* | 0.02 | 0.16 |
| 79834 | *PEAK1* | 0.09 | 0.30 |
| 5747 | *PTK2* | 0.04 | 0.31 |
| 5908 | *RAP1B* | 0.02 | 0.49 |
| 7050 | *TGIF1* | 0.01 | 0.44 |
| 84617 | *TUBB6* | 0.05 | 0.93 |
| 8061 | *FOSL1* | 0.11 | 0.16 |
| 6615 | *SNAI1* | 0.36 | 0.33 |
| 4323 | *MMP14* | 0.25 | 0.32 |

FC: fold change
