# Supplementary figures and images for: High expression of Collagen Triple Helix Repeat Containing 1 (CTHRC1) facilitates progression of oesophageal squamous cell carcinoma through MAPK/MEK/ERK/FRA-1 activation
Source: J Exp Clin Cancer Res. 2017 Jun 23;36:84. doi: 10.1186/s13046-017-0555-8 (PMC5481965; doi:10.1186/s13046-017-0555-8)

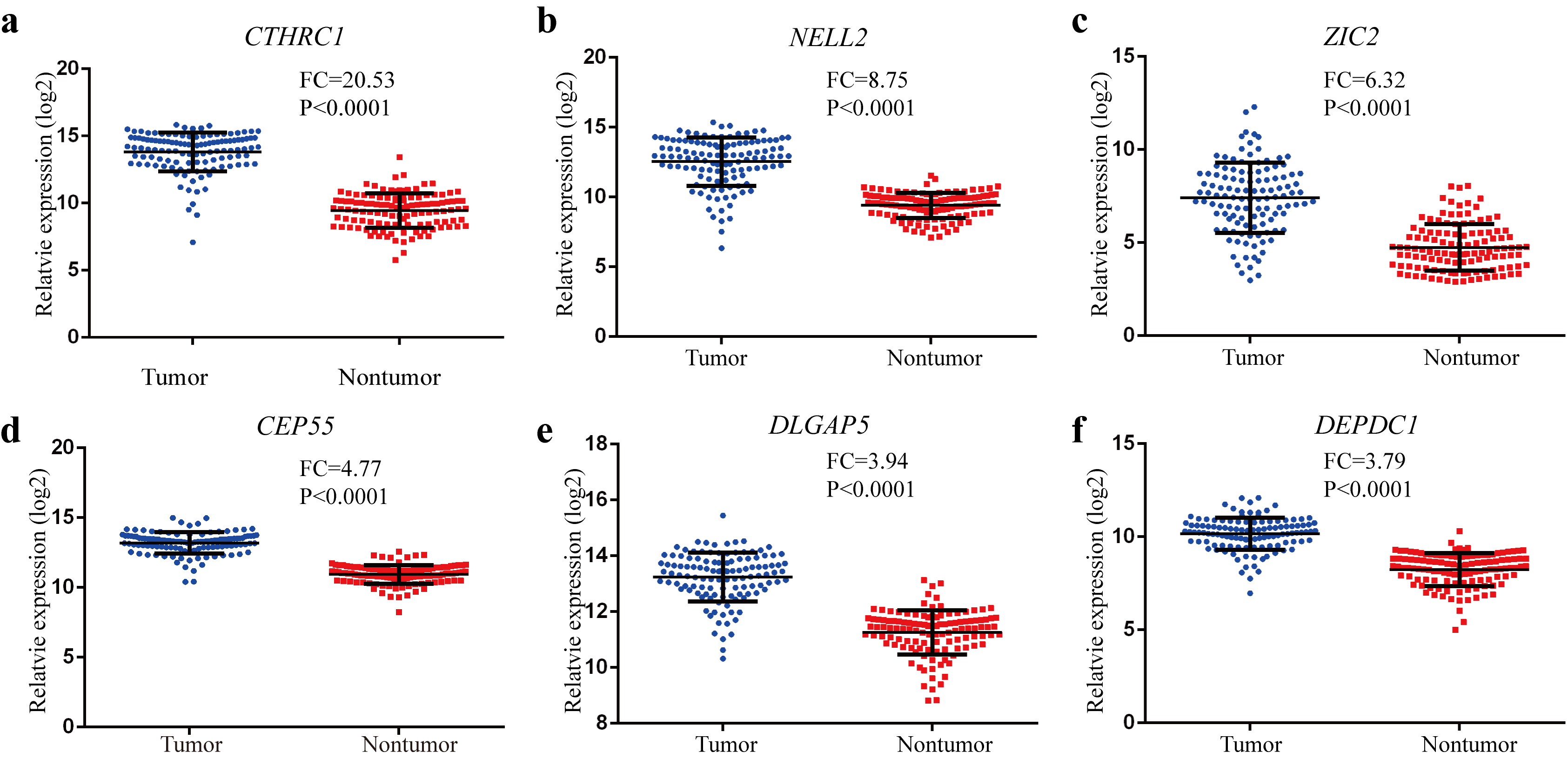

Supplement: Supplementary file 3 — Analysis of Collagen triple helix repeat containing-1 (CTHRC1), Neural EGFL like 2 (NELL2), DLG associated protein 5 (DLGAP5), DEP domain containing 1 (DEPDC1), Zic family member 2 (ZIC2) and Centrosomal protein 55 (CEP55) mRNA levels according to previous transcriptome-wide microarray profiling data (n = 119). The P value was generated by Wilcoxon test. FC: Fold change. (TIF 554 kb) [file 13046_2017_555_MOESM3_ESM.tif]

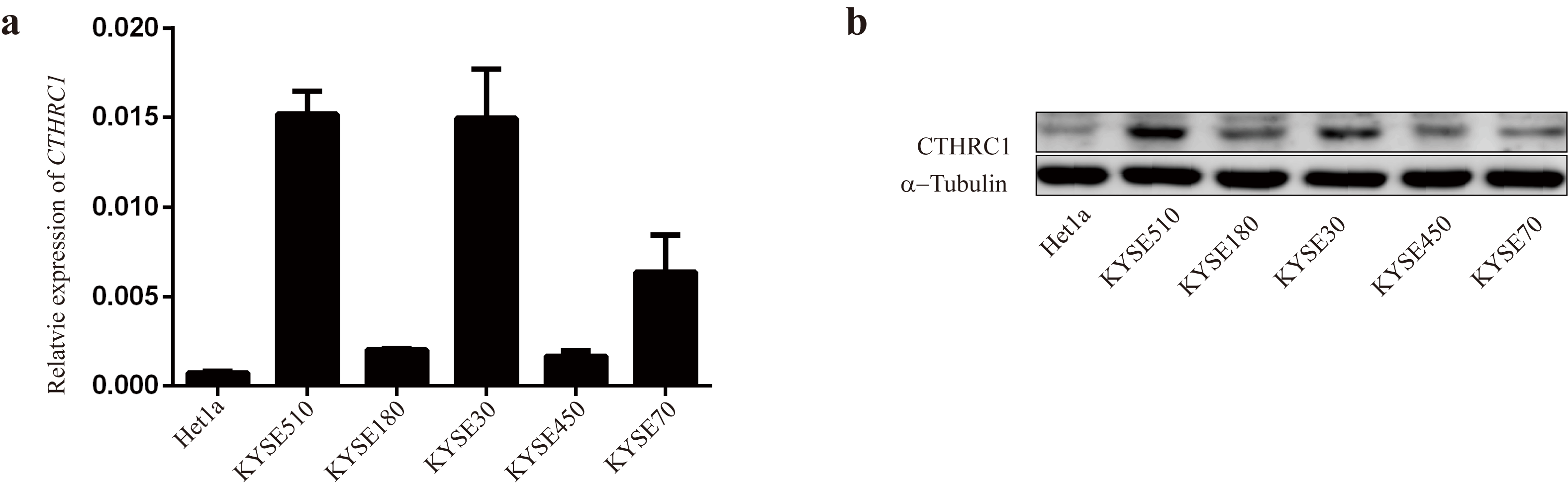

Supplement: Supplementary file 4 — Expression of CTHRC1 in ESCC cell lines was analysed by RT-PCR (a) and western blot analysis (b). (TIF 488 kb) [file 13046_2017_555_MOESM4_ESM.tif]

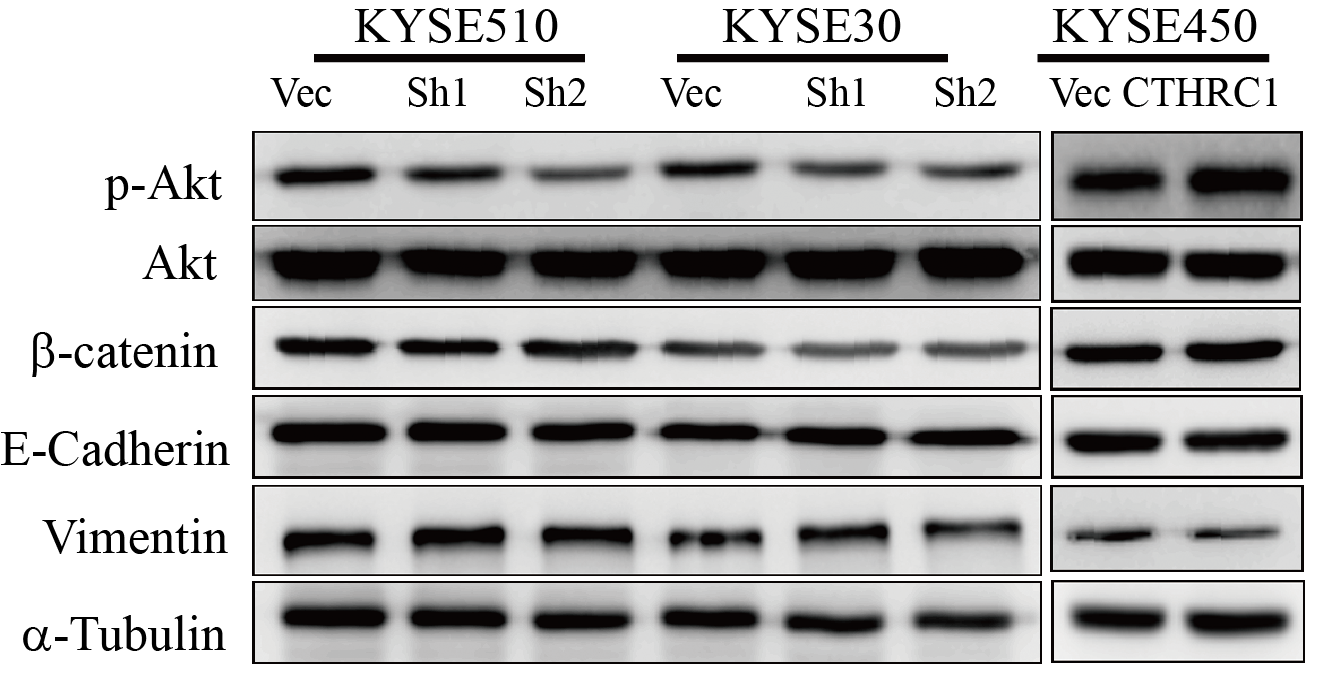

Supplement: Supplementary file 5 — The levels of p-Akt, Akt, β-catenin, E-cadherin and vimentin proteins were determined by western blot in KYSE510 and KYSE30 cells depleted for CTHRC1 expression and KYSE450 cells overexpressing CTHRC1 as well as corresponding control cells. (TIF 670 kb) [file 13046_2017_555_MOESM5_ESM.tif]
